# Effects of a 16-week recreational small-sided games soccer intervention on body composition and physical fitness in sedentary young adults: A randomized controlled study

**DOI:** 10.1016/j.heliyon.2024.e25242

**Published:** 2024-01-29

**Authors:** Qi Xu, Kai Qi, Guiyang Liu, TingYu Li, Filipe Manuel Clemente

**Affiliations:** a Gdansk University of Physical Education and Sport, 80-336, Gdańsk, Poland; b Physical Education and Health Education, Udon Thani Rajabhat University 64 Thaharn Road, Muang, Udon Thani, 41000, Thailand; c Research Center in Sports Performance, Recreation, Innovation and Technology (SPRINT), 4960-320, Melgaço, Portugal; d Escola Superior Desporto e Lazer, Instituto Politécnico de Viana Do Castelo, Rua Escola Industrial e Comercial de Nun’Álvares, 4900-347, Viana Do Castelo, Portugal

**Keywords:** Football, Physical fitness, Physical education, Body composition, Recreational football, Small-sided games, Physical exercise

## Abstract

Recreational small-sided games (SSGs) have demonstrated positive effects on body composition and physical fitness, while minimizing adverse outcomes. In this randomized controlled study, we aimed to investigate the impact of incorporating an additional 16-week intervention program involving recreational soccer SSGs on parameters related to body composition and physical fitness in sedentary young adult males and females. Sixty sedentary participants, with a mean age of 20.2 years, were randomly assigned to either the small-sided games group (SSG; n = 30) or the active control group, which participated in regular physical education classes (CG; n = 30). The SSG group engaged in the same activities as the control group but additionally participated in a recreational SSG football program. This program involved continuous and intermittent moderate-to high-intensity exercises conducted on 20 m × 30 m and 30 m × 50 m football fields. In contrast, the CG group received 1 h of physical education once a week. The interventions were administered for a duration of sixteen weeks. Baseline, 8-week, and post-intervention assessments were conducted to measure body mass (BM), body mass index (BMI), waist circumference (WC), hip circumference (HC), waist-to-hip ratio (WHR), skinfold thickness (SFT), standing broad jump (SBJ), vertical jump (VJ), handgrip strength (HG) for both left and right hands, shuttle run distance (SRD), and shuttle run estimated VO2max. Results indicated that both male and female participants in the SSG group exhibited significant improvements in BM, BMI, SFT, WC, HC, and WHR following the intervention (p < 0.05), whereas the control group demonstrated no significant changes over the study period (p > 0.05). Additionally, SSG participants (regardless of sex) displayed significant enhancements in SBJ, VJ, HG, SRD, and VO2max (p < 0.05), while the control group did not exhibit any significant alterations (p > 0.05). The findings from this experimental study suggested that a 16-week recreational soccer SSG intervention effectively enhanced body composition and physical fitness among overweight sedentary young adults, offering a pleasurable alternative to conventional training approaches.

## 1 Introduction

The prevalence of sedentary behavior among young adults has become a growing concern on a global scale, as evidenced by numerous scientific reports and findings [1]. Sedentary lifestyles, characterized by prolonged periods of sitting or low levels of physical activity, have become increasingly prevalent in this demographic [2]. Research consistently highlights the detrimental consequences of sedentarism, demonstrating a strong association with an elevated risk of non-communicable diseases [3]. Studies indicate that sedentary behavior is linked to an increased likelihood of developing conditions such as cardiovascular diseases [4], diabetes, and obesity [5]. Furthermore, a sedentary lifestyle is intricately connected to a decrease in fitness levels and overall health [6]. The lack of physical activity contributes to reduced cardiovascular fitness, muscle strength, and flexibility, adversely affecting the physiological well-being of young adults [3]. Addressing the sedentary epidemic through evidence-based interventions is crucial to mitigate the risk of non-communicable diseases and promote overall health in this population [7].

Scientific evidence consistently underscores the efficacy of physical exercise, particularly intense intermittent exercise, in enhancing physical fitness and positively influencing body composition [8]. High-intensity intermittent exercise, such as interval training, has been shown to significantly improve cardiovascular fitness [9], increase muscle strength, and enhance metabolic function [10]. These adaptations not only counteract the detrimental effects of sedentary behavior but also contribute to favorable changes in body composition by reducing body fat and increasing lean muscle mass [11].

Scientific evidence highlights the transformative potential of recreational soccer (as a form of intermittent intense exercise) in community programs as a compelling strategy to counter sedentary behavior while promoting health and fitness [12] (ref). Participating in recreational soccer not only engages sedentary populations in enjoyable physical activity but also fosters a social environment, addressing key factors that contribute to sustained participation [13]. Research indicates that recreational soccer, with its dynamic nature and intermittent bursts of high-intensity effort, mirrors the benefits of structured high-intensity interval training [14]. This form of exercise has been associated with improved cardiovascular fitness [12], enhanced metabolic health [15], and favorable body composition changes [16]. Moreover, the communal and enjoyable aspects of soccer contribute to increased adherence to physical activity, positively impacting mental well-being and overall quality of life [17]. By incorporating recreational soccer into community programs, evidence-based approaches align with both the physiological and social dimensions, offering a holistic solution to combat sedentary lifestyles and promote lasting health benefits [18].

Current scientific findings underscore the efficacy of recreational soccer in positively influencing body composition and physical fitness across diverse age groups, particularly in young [19] and older populations [20]. However, there exists a notable gap in our understanding of the impact of recreational soccer on body composition and physical fitness in the critical transitional phase of young adulthood, characterized by the shift from high school to university. This demographic is particularly susceptible to declines in physical activity, potentially exacerbating sedentary behavior [21]. While the benefits of recreational soccer are well-established in other age brackets, further research is warranted to comprehensively elucidate its effects on body composition and physical fitness in young adults during this crucial life transition. Closing this knowledge gap could inform targeted interventions to mitigate sedentary trends and optimize health outcomes in this specific population.

To address this gap in the literature, the present study aimed to investigate the potential benefits of a 16-week recreational soccer small-sided games (SSGs) program on body composition-related parameters and physical fitness in sedentary young adult males and females. A control group, only enrolled in one physical education class a week, was also included for comparison purposes. Building on prior research [22,23], our hypothesis posited that individuals exposed to recreational soccer SSGs experienced a significant improvement in body mass index, fat mass, endurance performance, and neuromuscular performance compared to the control group.

## 2 Methods

### 2.1 Experimental design

This investigation utilized a randomized controlled two-arm study design and lasted for 16 weeks. Evaluations were conducted at baseline, the midpoint (8th week), and the end of the 16 weeks. This study employed a per protocol analysis, which considered attendance at all evaluations and sessions, as well as non-enrollment in specific diets. Initially, 10 universities were contacted based on geographic location, and seven of them agreed to participate in the study. All students who met the inclusion criteria were invited to participate, resulting in a participation rate above 85 %. The final participants were registered in the system, the experiment was carried out in January 2023 and data collection for all participants was completed in the end of April 2023. The researchers ensured the accuracy of the experiment by using the same test methods and tools across all participating schools. The participants were randomly assigned to either the SSGs group or the control group using a simple randomization process involving the use of letters. Allocation concealment was rigorously ensured through centralized randomization and sealed envelopes, bolstering the reliability and impartiality of treatment assignments in the experiment. The procedure was carried out by an assessor who was blinded to the experiment.

Participants were mandated to attend training sessions held at the specified experimental site, facilitated by the research team. Moreover, participants were directed to adhere to a dietary regimen tailored for obese individuals throughout the study. It is noteworthy that neither participants, assessors, nor instructors were blinded to the experiment.

### 2.2 Participants

Recruitment involved reaching out through social media and networks, where individuals expressing interest voluntarily participated in the experiment. To accommodate potential dropouts and enhance sensitivity to smaller differences, a total of 84 subjects were recruited. Of these, 8 participants did not meet the eligibility criteria, and 16 participants failed to complete the training. Ultimately, 60 participants completed the entire experiment, with 30 in the SSGs group and 30 in the control group.

**Fig. 1:**
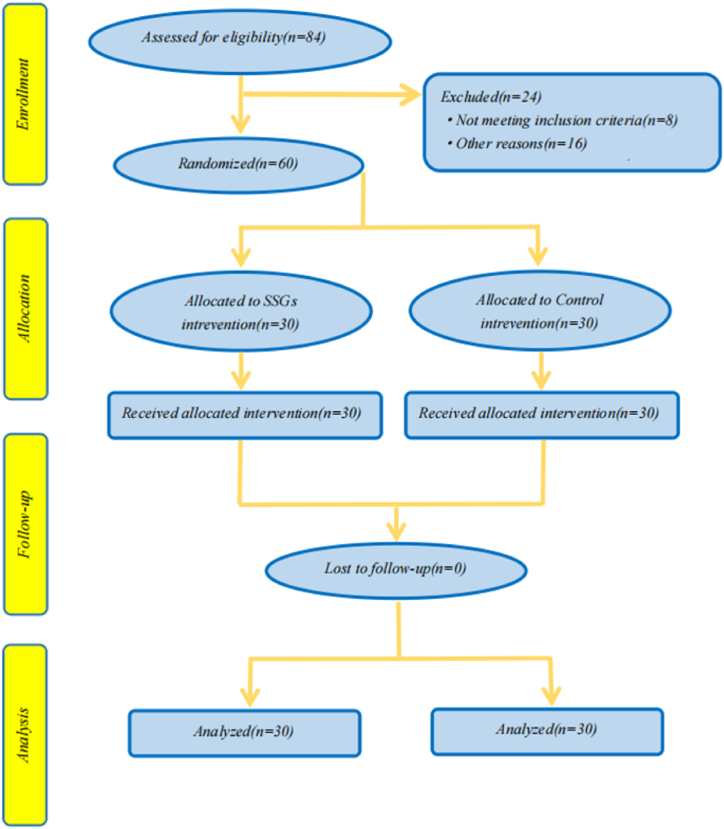
Flowchart of participant selection. SSGs: small-sided games, football-themed recreational small side games. Control: normal diet and participation in normal physical education control group.

The study comprised university students residing in diverse urban areas of Nanchang, China, an eastern city in the country. Inclusion criteria mandated that participants be sedentary men or women with overweight or obesity (BMI >28) and devoid of other health issues. Attendance at all three assessments (anthropometry, cardiovascular, and musculoskeletal fitness), maintenance of a training program adherence exceeding 75 %, and non-enrollment in additional nutritional or pharmacological strategies for weight loss were also prerequisites. Detailed participant characteristics are provided in Table 1, while the participant enrollment and retention process is delineated in Fig. 1.

**Table 1:** Characteristics of the study participants.

	All	Men	Women
N	60	30	30
Age (years)	20.2 ± 1.0	20.1 ± 0.8	20.3 ± 1.1
Height (m)	1.67 ± 0.06	1.72 ± 0.04	1.62 ± 0.04
Body mass (kg)	86.3 ± 11.8	94.4 ± 9.7	78.2 ± 7.3
Body mass index (kg/m^2^)	30.4 ± 2.5	31.3 ± 2.7	29.5 ± 1.8
Waist Circumference (cm)	99.7 ± 6.5	100.0 ± 6.5	99.4 ± 6.6
Hip Circumference (cm)	106.4 ± 5.5	108.0 ± 5.9	104.9 ± 4.6
Waist-to-hip ratio (%)	93.4 ± 5.0	92.3 ± 4.1	94.5 ± 5.6
Skin fold thickness (mm)	58.8 ± 4.3	59.1 ± 4.5	58.5 ± 4.0
Education level	undergraduates education		

m: meters; kg: kilograms; cm: centimeters; %: percentage; mm: millimeters.

Prior to participation, every participant received comprehensive information about any potential risks or discomfort associated with the experiment. Upon agreement to participate, the guardians provided their consent by signing a consent form. The study adhered to ethical standards for medical research involving human subjects as outlined in the Declaration of Helsinki. Ethical approval for the study was obtained from the Institutional Ethical Review Board of Chengdu Institute of Physical Education, with reference code 2023#104.

### 2.3 Methodological procedures

In the initial session, demographic data were collected, and participants' physical activity levels were assessed using the short form of the International Physical Activity Questionnaire [24] as a secondary outcome. Sedentary levels were determined based on participants' scores.

Furthermore, throughout the study (during the 3rd, 6th, and 9th weeks), participants were closely monitored to assess their physical activity (PA) levels. This monitoring aimed to ensure that they were not engaging in activities beyond the scope of the expected ones. Upon conducting exploratory analysis, no discernible differences were observed in the behaviors of participants, both within the experimental and control groups, even after adjusting for the implemented training programs.

In the second evaluation phase, a battery of tests assessing anthropometric measurements, body composition, and physical fitness was administered by a team of assessors. The team comprised four sports scientists, each holding at least a master's degree and possessing extensive experience in physical fitness and anthropometry assessments.

The initial anthropometric and physical fitness test occurred at 3:00 p.m. on December 8, 2022, in the gymnasium of the author's workplace, with a temperature of 5 °C and relative humidity at 53 %. Anthropometric measurements were initiated, with participants measured in the order of their registration. Subsequently, the physical fitness tests began 10 min after anthropometry, following this sequence: (i) handgrip strength test; (ii) standing broad jump; (iii) vertical jump tests; and (iv) the 20-m shuttle run test. Three minutes of rest interspersed between each of the physical fitness tests. Baseline testing occurred at the 8th and 16th weeks at the same location as the initial test.

#### 2.3.1 Anthropometry and body composition

Anthropometric measurements were conducted on participants following standardized procedures. Stature was measured using the Seca digital weighing scales stadiometer (803, Seca, China) with a technical error of ±0.1 kg. Measurements were taken with participants barefoot and wearing regular shirts and shorts. Body mass was measured using the Smart Body Analyzer (WS-50, Withings, France) with a technical error of ±0.5 cm. The body mass index (BMI) was subsequently calculated using the measured values of stature and body mass.

A highly trained and standardized technician, following recommended protocols, measured skinfold thickness [25]. Subscapular, suprailiac, triceps, biceps, and thigh skinfold measurements were assessed. The caliper (Lange Skinfold Caliper, Beta Technology Incorporated, Cambridge, MD) was applied 1 cm below the thumb and finger holding the fold, and thickness was recorded to the nearest 0.1 cm. All skinfold measurements were taken three times, and the average was recorded [26].

Waist circumference was measured by identifying the top of the hip bone and the bottom of the ribcage, measuring at the level midway between these landmarks. Hip circumference was measured at the widest part of the hips, typically at the level of the greater trochanters. In summary, the primary outcomes considered for the current study encompassed body mass, BMI, waist circumference, hip circumference, waist-to-hip ratio, and skinfold thickness.

Under professional guidance, stature, body mass, waist circumference, and hip circumference were measured once, and skinfold thickness was measured three times. A single researcher, with expertise in anthropometric assessment and a demonstrated high level of reliability (±0.5 cm), performed all measurements. The MPS Micro rope displacement sensor (XXS, MILONT, China) with a technical error of ±0.1 cm was used by the researcher to ensure precision in the measurement process.

#### 2.3.2 20-m shuttle run test

The 20-m shuttle run test was administered to participants following the original protocol previously published [27]. Participants were directed to run back and forth between two lines placed 20 m apart, pivoting around a central point situated between the two lines. Instead of crossing the line before turning, participants had the option to spin and continue running within the boundaries of the two lines. The test concluded if the participant failed to reach a line after two consecutive beeps or if the participant neglected to wait for the beep twice in a row. The total distance covered by each participant was recorded as the primary outcome. Additionally, VO2 max was estimated using the equation VO2max = −24.4 + 6.0 final velocity achieved. It is noteworthy that the 20-m shuttle run, also recognized as the Buzzer test, has been validated as a reliable tool for predicting the maximal oxygen uptake (VO2max) and is widely acknowledged as the gold standard for field-based assessments of aerobic fitness [28]. The primary outcomes for the current research included the shuttle run distance and estimated VO2max.

#### 2.3.3 Handgrip dynamometry

After comparing the Jamar, DynEx, and TKK precise dynamometers, handgrip strength was evaluated using the TKK dynamometer (TKK 5101 Grip-D, Takey, Tokyo, Japan), identified as the most accurate among the three. In terms of criterion-related validity, the Jamar and DynEx dynamometers were found to underestimate handgrip strength levels by −192 and 21.43 kg, respectively. The TKK dynamometer exhibited the lowest level of systematic error (0.49 kg) and is thus deemed the most appropriate tool for assessing handgrip strength within the studied population [29].

Participants were instructed to extend their arms, using their dominant hand to exert maximal force on the dynamometer. The grip had to be sustained for at least 2 s, and the task was repeated twice, with the peak force recorded for analysis. Each participant underwent 3 trials per hand, with a 2-min rest between trials and a 3-min rest between hands. The trial producing the highest value was utilized for statistical analysis. The average coefficient of variation for the within-participant (variability between trials) analysis was 2.1 % in the handgrip strength. The primary outcomes for the current research involved the maximal handgrip strength in both left and right hands.

#### 2.3.4 Standing broad jump and vertical jump

The My Jump 2 app on iPhone X was employed to calculate jump height by manually selecting the take-off and landing frames of the video. The app utilizes the equation:

(1) h=t2×1.22625

as described by Bosco et al. [30], where h represents jump height (in meters) and t denotes flight time (in seconds). My Jump 2 was employed to measure jumping performance, as it has been demonstrated to be a valid and reliable tool for assessing both jump height and jump length [31].

For the vertical jump, participants were instructed to initiate the jump [32] from a position of 90° knee flexion [33], with feet shoulder-width apart and hands on their hips. They were directed to jump for maximum height while keeping their hands on their hips [34]. In the standing broad jump, participants were positioned on the start line and instructed to jump horizontally as far as possible while maintaining hands on hips. Swinging arms during the jump was not permitted. Successful jumps required participants to land on both feet without secondary corrective motions [35]. Each participant performed both jumps three times, with a 2-min rest between trials and a 3-min rest between jump tests. The trial producing the highest value was used for statistical analysis. The average coefficient of variation for the within-participant (variability between trials) analysis was 3.4 % in the vertical jump and 3.7 % in the standing broad jump.

The primary outcomes for the current research included the highest vertical and horizontal jumps.

### 2.4 Training intervention

The SSGs intervention consisted of five weekly sessions held over the period of experiment, each lasting 60 min. This additional training was incorporated alongside the participants' regular 1-h weekly physical education. The control group, in contrast, did not undergo any specific training and continued with their regular daily activities, including a standard diet and 1 h of weekly physical education.

SSGs training sessions occurred on the school football field after regular class hours. A 10-min warm-up preceded 40 min of small-sided games and technical drills, concluding with 10 min of cool-down exercises. To ensure consistent training intensity and participant safety, each individual wore a heart rate monitor (Polar RS400, Kempele, Finland) during every session. Participant maximal heart rate (HRmax) was calculated using the Ruffier test [36], and two college physical education teachers, under the author's guidance, assisted in conducting the experiment.

Exercise perceived intensity was assessed using the Borg CR 10 Scale and the Talk Test [37]. Additionally, participants completed a personal exercise monitoring card after each session to track health status and exercise levels. The card included the date, type, and duration of the recreational soccer training, subjective assessment of exercise intensity (Borg CR 10 Scale), physical health before and after training, rest time post-training, and reasons for any missed sessions.

The SSGs intervention targeted major muscle groups and cardiopulmonary function with exercises such as shuttle runs, sprints, passing, jumps, dribbling, and shooting. For the initial eight weeks, the exercise-to-rest ratio was set at 5:1 based on individual athletic ability and training progress. At the midpoint of the 16-week intervention, we assessed participants' exercise levels and physical health, adjusting the exercise-to-rest ratio and training type for the subsequent eight weeks accordingly. Table 2 provides a detailed breakdown of workout and rest intervals, along with the type of training, during the intervention. The participants in the SSG group were assigned to teams based on their proficiency levels, with adjustments made as necessary to ensure the games remained as competitive as possible. The selection process was overseen by the trainer in charge, who has experience in community programs and soccer. The games were organized by sex, with men competing against men and women facing off against women.

**Table 2:** The characteristics of small-sided games intervention.

Week number	Days Per Week	Work Duration (min)	Time of Rest Interval (min)	Number of Exercises	Time between Sets (min)	Training Drills
1	5	10	2	3	2	3V3 fun games on a 30 m × 20 m field, set up a 0.5 m high net in the middle of the pitch (football tennis)
2	5	10	2	3	2	3V3 competition on a 30 m × 20 m field
3	5	15	3	2	3	5V5 fun games on a 30 m × 20 m field (Passing theme)
4	5	15	3	2	3	5V5 race on a 30 m × 20 m field, place hurdles on the field
5	5	15	3	2	3	3V3 fun games on a 30 m × 20 m field, dribble according to the instructions
6	5	25	5	1	/	A relay race of 5V5 dribbling over obstacles on a 30 m × 20 m field
7	5	25	5	1	/	3V3 Competition at 30 m × 20 m field
8	5	15	3	2	3	5V5 Competition at 30 m × 20 m field
9	5	20	4	2	2	5V5 fun games on a 50 m × 30 m field
10	5	20	4	2	2	3V3 competition on a 50 m × 30 m field
11	5	20	4	2	2	5V5 fun games on a 50 m × 30 m field
12	5	25	5	1	/	3V3 fun games on a 50 m × 30 m field
13	5	25	5	1	/	3V3 recreational Football Endurance Training on 50 m × 30 m Field
14	5	25	5	1	/	3V3 competition in a 50 m × 30 m field
15	5	15	3	2	3	5V5 fun games on a 50 m × 30 m field
16	5	15	3	2	3	5V5 competition on a 50 m × 30 m field

Min: minutes.

The SSGs intervention comprised a total of 80 sessions conducted at the school's football stadium, where the author is employed. These sessions occurred on Mondays, Wednesdays, and Thursdays from 4:30 p.m. to 5:40 p.m., and on Fridays and Saturdays from 6:30 p.m. to 7:40 p.m. Throughout each session, the participants' physical condition and movement status were diligently monitored. The author supervised the implementation of the training program, with two additional teachers offering technical corrections and encouragement to the participants.

The percentage of adherence in the SSG interventions was 90.0 ± 1.0 % in men (91 % between the first and second assessment and 89 % between the second and third assessment), while it was 89.5 ± 2.5 % in women (92 % between the first and second assessment and 87 % between the second and third assessment).

### 2.5 Statistical procedures

The study's sample size was determined utilizing G*Power 3.1 software (Düsseldorf, Germany), specifically selecting the ANOVA: Repeated measures, Within-between interaction option. Power, α, and effect size were set at 0.8, 0.25, and derived from previous studies [38,39]. Results revealed that the overall effect size of Matrix Factorization on the competitive ability of football players predominantly falls within the range of 0.50–2.37, with an average effect size of 0.84. A minimum of 22 participants per group was deemed necessary for this study.

Descriptive statistics are presented as mean and standard deviation. Normality and homogeneity were assessed using the Kolmogorov-Smirnov and Levene's tests, respectively. The Smallest Worthwhile Change (SWC) was determined using Cohen's effect size principle, a measure that indicates the magnitude of change deemed meaningful. The formula utilized was:

(2) $$0.2\times \sqrt{\frac{{SD}_{1}^{2}+{SD}_{3}^{2}}{2}}$$,

where ${SD}_{1}^{2}$ and ${SD}_{3}^{2}$ represent the standard deviations of the measurements for the “1st” and “3rd” tests, respectively. This calculation was performed for each variable, considering a sample size of 30 participants. The resulting SWC values represent the smallest change in each variable that is considered meaningful within the context of the study.

Once normality and homogeneity were confirmed (p > 0.05), an independent *t*-test and Cohen's d effect size test were performed to compare the baseline levels between groups. A mixed ANOVA (times * group) was used to examine interactions between factors, with partial eta squared used to estimate effect sizes. Pairwise comparisons were analyzed using Bonferroni's post hoc test. Cohen's d was also calculated to determine the magnitude of differences in pairwise comparisons. All statistical analyses were performed using SPSS software (version 28.0.0.0, IBM, Chicago, USA) with a significance level set at p < 0.05.

## 3 Results

### 3.1 Baseline assessments

In men, there were no significant differences between groups in the baseline levels of body mass (p = 0.649; d = 0.168), body mass index (p = 0.330; d = 0.362), waist circumference (p = 0.888; d = 0.052), hip circumference (p = 0.719; d = 0.133), waist-to-hip ratio (p = 0.087; d = 0.648), skinfold thickness (p = 0.459; d = 0.274), standing broad jump (p = 0.065; d = 0.701), vertical jump (p = 0.072; d = 0.682), shuttle run distance (p = 0.096; d = 0.629), and shuttle run estimated VO2max (p = 0.105; d = 0.612). However, significant differences were found in handgrip strength for the right (p = 0.009; d = 1.028) and left (p = 0.017; d = 0.926) hands.

In women, there were no significant differences between groups in the baseline levels of body mass (p = 0.680; d = 0.152), body mass index (p = 0.495; d = 0.252), waist circumference (p = 0.628; d = 0.179), hip circumference (p = 0.593; d = 0.197), waist-to-hip ratio (p = 0.292; d = 0.392), skinfold thickness (p = 0.270; d = 0.411), standing broad jump (p = 0.051; d = 0.746), vertical jump (p = 0.505; d = 0.247), handgrip strength for the right (p = 0.058; d = 0.721) and left (p = 0.094; d = 0.632) hands, shuttle run distance (p = 0.512; d = 0.243), and shuttle run estimated VO2max (p = 0.544; d = 0.224).

### 3.2 Anthropometry and body composition

Table 3 presents the descriptive statistics of participant anthropometry and characteristics across the three assessment time points in the experimental study. Supplementary Material 1 illustrates the variation among participants (within-participation variation) across assessments, taking into account anthropometric and body composition variables. No significant interactions on body mass (F = 1.833; p = 0.170; $\eta_{p}^{2}$ = 0.063), body mass index (F = 0.878; p = 0.421; $\eta_{p}^{2}$ = 0.031), waist circumference (F = 1.634; p = 0.204; $\eta_{p}^{2}$ = 0.056), hip circumference (F = 1.726; p = 0.187; $\eta_{p}^{2}$ = 0.059), while revealed significant interactions on waist-to-hip circumference (F = 3.916; p = 0.026; $\eta_{p}^{2}$ = 0.125) and skinfold thickness (F = 3.721; p = 0.031; $\eta_{p}^{2}$ = 0.119) were found interacting sex, group and time.

**Table 3:** Descriptive statistics, presented as mean ± standard deviation, used to report participant characteristics across the three assessment time points in the experimental study.

	Men SSGs 1stA	Men SSGs 2ndA	Men SSGs 3rdA	Within-SSGs group change 3rd−1st	SWC 3rd−1st	Men Control 1stA	Men Control 2ndA	Men Control 3rdA	Within-SSGs group change 3rd−1st	SWC 3rd−1st
Body mass (kg)	93.60 ± 10.22^¶,@^	87.53 ± 9.54^#,@^	79.86 ± 9.02^#,¶^	−14.66 % |d = −1.079	1.93	95.26 ± 9.60	95.60 ± 8.74	95.00 ± 8.29	−0.27 % |d = −0.028	0.32
BMI (kg/m^2^)	31.86 ± 2.97^¶,@^	30.00 ± 3.09^#,@^	27.13 ± 2.87^#,¶^	−14.88 % |d = −1.286	0.61	30.86 ± 2.53	31.13 ± 2.47	31.13 ± 2.72	0.87 % |d = 0.106	0.25
Waist circunference (cm)	98.66 ± 6.23^¶,@^	92.40 ± 5.76^#,@^	84.40 ± 6.43^#,¶^	−14.50 % |d = −1.059	1.35	98.33 ± 6.64	98.00 ± 5.43	97.33 ± 5.51	−1.02 % |d = −0.150	0.42
Hip circunference (cm)	108.40 ± 5.77^¶,@^	101.13 ± 4.79^#,@^	94.13 ± 5.12^#,¶^	−13.18 % |d = −1.094	1.26	107.60 ± 6.26	107.20 ± 5.20	106.53 ± 5.47	−1.00 % |d = −0.162	0.38
Waist-to-hip ratio (cm)	93.66 ± 4.57^¶,@^	91.33 ± 5.05^#,@^	89.20 ± 4.93^#,¶^	−4.77 % |d = −0.745	0.96	91.06 ± 3.34	91.26 ± 2.91	91.26 ± 2.57	0.22 % |d = 0.075	0.13
Skinfold thickness (mm)	59.73 ± 4.80^¶,@^	53.33 ± 3.77^#,@^	48.20 ± 4.41^#,¶^	−19.36 % |d = −1.795	1.15	58.46 ± 4.42	58.00 ± 4.00	57.06 ± 4.04	−2.39 % |d = −0.334	0.34
	Women SSGs 1stA	Women SSGs 2ndA	Women SSGs 3rdA			Women Control 1stA	Women Control 2ndA	Women Control 3rdA		
Body mass (kg)	78.80 ± 4.34^¶,@^	74.33 ± 4.30^#,@^	65.26 ± 4.90^#,¶^	−17.18 % |d = −1.365	1.06	77.66 ± 9.59	77.93 ± 9.39	77.20 ± 8.85	−0.59 % |d = −0.048	0.21
BMI (kg/m^2^)	29.73 ± 1.90^¶,@^	28.13 ± 1.80^#,@^	24.86 ± 1.80^#,¶^	−16.35 % |d = −1.487	0.47	29.26 ± 1.79	29.26 ± 1.90	28.73 ± 1.90	−1.81 % |d = −0.361	0.22
Waist circunference (cm)	98.80 ± 5.54^¶,@^	93.66 ± 4.91^#,@^	84.00 ± 4.27^#,¶^	−14.97 % |d = −1.206	1.05	100.00 ± 7.70	99.20 ± 7.33	98.00 ± 7.37	−2.00 % |d = −0.254	0.39
Hip circunference (cm)	105.40 ± 3.97^¶,@^	100.06 ± 3.75^#,@^	91.46 ± 4.40^#,¶^	−13.20 % |d = −1.118	1.07	104.46 ± 5.38	103.60 ± 4.67	102.66 ± 5.86	−1.72 % |d = −0.257	0.46
Waist-to-hip ratio (cm)	93.40 ± 6.23^¶,@^	93.86 ± 5.48^#,@^	91.53 ± 4.89^#,¶^	−1.99 % |d = −0.304	0.70	95.60 ± 4.91	95.60 ± 4.92	95.40 ± 5.50	−0.21 % |d = −0.046	0.13
Skinfold thickness (mm)	57.73 ± 3.84^¶,@^	52.13 ± 3.56^#,@^	43.73 ± 4.33^#,¶^	−24.28 % |d = −2.09	1.01	59.46 ± 4.56	58.46 ± 4.62	57.33 ± 5.40	−3.57 % |d = −0.397	0.39

A: assessment; SWC: smallest worthwhile change; #: significantly different from 1stA at p < 0.05; ¶: significantly different from 2ndA at p < 0.05; @; significantly different from 3rdA at p < 0.05.

No significant interactions (time*sex) were found on body mass (F = 0.850; p = 0.403; $\eta_{p}^{2}$ = 0.015), body mass index (F = 1.946; p = 0.154; $\eta_{p}^{2}$ = 0.034), waist circumference (F = 1.526; p = 0.225; $\eta_{p}^{2}$ = 0.027), hip circumference (F = 0.748; p = 0.453; $\eta_{p}^{2}$ = 0.013), waist-to-hip ratio (F = 2.467; p = 0.107; $\eta_{p}^{2}$ = 0.042), while significant interactions were found on skinfold thickness (F = 4.391; p = 0.019; $\eta_{p}^{2}$ = 0.073).

Significant interactions (time*group) were found on body mass (F = 192.648; p < 0.001; $\eta_{p}^{2}$ = 0.775), body mass index (F = 147.904; p < 0.001; = 0.725), waist circumference (F = 206.746; p < 0.001; $\eta_{p}^{2}$ = 0.787), hip circumference (F = 124.738; p < 0.001; $\eta_{p}^{2}$ = 0.690), waist-to-hip ratio (F = 13.127; p < 0.001; $\eta_{p}^{2}$ = 0.190) and skinfold thickness (F = 142.838; p < 0.001; $\eta_{p}^{2}$ = 0.718).

### 3.3 Physical fitness

Table 4 presents the descriptive statistics of participant physical fitness across the three assessment time points in the experimental study. Supplementary Material 2 illustrates the variation among participants (within-participation variation) across assessments, taking into account physical fitness variables. No significant interactions on standing broad jump (F = 0.132; p = 0.976; $\eta_{p}^{2}$ = 0.005), vertical jump (F = 0.550; p = 0.580; $\eta_{p}^{2}$ = 0.020), handgrip right (F = 0.730; p = 0.486; $\eta_{p}^{2}$ = 0.026), handgrip left (F = 0.262; p = 0.771; $\eta_{p}^{2}$ = 0.009), shuttle run distance (F = 1.526; p = 0.226; $\eta_{p}^{2}$ = 0.053) and shuttle run estimated VO2max (F = 1.601; p = 0.211; $\eta_{p}^{2}$ = 0.055) were found between sex, group and time.

**Table 4:** Descriptive statistics, presented as mean ± standard deviation, used to report participant characteristics across the three assessment time points in the experimental study.

	Men SSGs 1stA	Men SSGs 2ndA	Men SSGs 3rdA	Within-SSGs group change 3rd−1st	SWC 3rd−1st	Men Control 1stA	Men Control 2ndA	Men Control 3rdA	Within-SSGs group change 3rd−1st	SWC 3rd−1st
Standing broad jump (cm)	173.3 ± 8.9^¶,@^	176.7 ± 8.9^#,@^	181.7 ± 8.3^#,¶^	4.84 % |d = 0.948	1.95	181.7 ± 14.3	181.4 ± 12.3	180.5 ± 11.5	−0.66 % |d = −0.086	1.29
Vertical jump (cm)	15.2 ± 4.7^¶,@^	18.5 ± 4.5^#,@^	22.3 ± 4.9^#,¶^	46.71 % |d = 1.217	2.38	19.5 ± 7.6	18.7 ± 5.5	18.9 ± 6.7	−2.95 % |d = −0.079	0.75
Handgrip left (kg)	33.2 ± 4.3^¶,@^	33.5 ± 4.5^#,@^	34.2 ± 4.7^#,¶^	3.01 % |d = 0.233	0.49	37.4 ± 4.6	37.0 ± 5.1	37.0 ± 5.2	−1.07 % |d = −0.092	0.32
Handgrip right (kg)	35.1 ± 4.4^¶,@^	35.8 ± 4.4^#,@^	36.5 ± 4.7^#,¶^	4.00 % |d = 0.291	0.72	39.9 ± 4.9	39.6 ± 5.4	39.0 ± 5.0	−2.26 % |d = −0.174	0.50
Shuttle run distance (m)	546.6 ± 153.3^¶,@^	650.6 ± 75.9^#,@^	745.3 ± 50.9^#,¶^	36.40 % |d = 1.254	128.87	468.0 ± 88.0	462.6 ± 84.8	476.0 ± 85.5	1.71 % |d = 0.088	8.20
Shuttle run estimated VO2max (ml/kg/min)	28.1 ± 3.2^¶,@^	30.3 ± 1.5^#,@^	32.2 ± 0.9^#,¶^	14.23 % |d = 1.305	2.52	26.5 ± 1.8	26.3 ± 1.7	26.7 ± 1.8	0.75 % |d = 0.055	0.14
	Women SSGs 1stA	Women SSGs 2ndA	Women SSGs 3rdA			Women Control 1stA	Women Control 2ndA	Women Control 3rdA		
Standing broad jump (cm)	159.8 ± 6.7^¶,@^	162.7 ± 6.6^#,@^	169.3 ± 4.8^#,¶^	5.95 % |d = 1.229	2.82	163.9 ± 3.8	163.9 ± 3.8	164.7 ± 4.5	0.49 % |d = 0.198	0.76
Vertical jump (cm)	8.9 ± 2.4^¶,@^	11.6 ± 2.8^#,@^	15.5 ± 3.9^#,¶^	74.16 % |d = 2.469	2.30	9.4 ± 1.6	9.2 ± 1.7	10.0 ± 1.9	6.38 % |d = 0.329	0.54
Handgrip left (kg)	23.1 ± 4.5^¶,@^	23.9 ± 4.5^#,@^	24.3 ± 4.1^#,¶^	5.19 % |d = 0.246	0.58	20.4 ± 3.9	20.7 ± 3.7	20.4 ± 3.2	0.00 % |d = 0.000	0.29
Handgrip right (kg)	25.3 ± 4.4^¶,@^	25.8 ± 4.2^#,@^	26.6 ± 4.2^#,¶^	5.14 % |d = 0.302	0.83	22.1 ± 4.2	22.1 ± 3.7	21.8 ± 3.2	−1.36 % |d = −0.158	0.26
Shuttle run distance (m)	300.0 ± 83.4^¶,@^	465.3 ± 69.8^#,@^	590.6 ± 45.2^#,¶^	96.88 % |d = 2.823	155.98	320.0 ± 81.4	330.7 ± 70.0	338.7 ± 66.1	5.84 % |d = 0.269	16.15
Shuttle run estimated VO2max (ml/kg/min)	23.0 ± 1.7^¶,@^	26.4 ± 1.5^#,@^	29.0 ± 0.9^#,¶^	26.09 % |d = 3.545	3.51	23.4 ± 1.7	23.6 ± 1.4	23.8 ± 1.4	1.71 % |d = 0.219	0.34

A: assessment; SWC: smallest worthwhile change; #: significantly different from 1stA at p < 0.05; ¶: significantly different from 2ndA at p < 0.05; @; significantly different from 3rdA at p < 0.05.

No significant interactions (time*sex) were found on standing broad jump (F = 2.406; p = 0.103; $\eta_{p}^{2}$ = 0.041), vertical jump (F = 0.116; p = 0.891; $\eta_{p}^{2}$ = 0.002), handgrip left (F = 2.892; p = 0.072; $\eta_{p}^{2}$ = 0.049), handgrip right (F = 0.437; p = 0.584; $\eta_{p}^{2}$ = 0.008) (F = 3.610; p = 0.041; $\eta_{p}^{2}$ = 0.061), while significant interactions were found on shuttle run distance (F = 3.610; p = 0.041; $\eta_{p}^{2}$ = 0.061) and shuttle run estimated VO2max (F = 3.862; p = 0.034; $\eta_{p}^{2}$ = 0.065).

Significant interactions (time*group) were found on standing broad jump (F = 66.542; p < 0.001; $\eta_{p}^{2}$ = 0.543), vertical jump (F = 47.201; p < 0.001; $\eta_{p}^{2}$ = 0.457), handgrip left (F = 13.927; p < 0.001; $\eta_{p}^{2}$ = 0.199), handgrip right (F = 30.327; p < 0.001; $\eta_{p}^{2}$ = 0.351), shuttle run distance (F = 67.986; p < 0.001; $\eta_{p}^{2}$ = 0.548) and shuttle run estimated VO2max (F = 66.325; p < 0.001; $\eta_{p}^{2}$ = 0.542).

## 4 Discussion

This study investigated the potential benefits of a 16-week recreational SSGs program on body composition and physical fitness among sedentary young adults. The primary outcome suggests that implementing this program holds promise for improving these parameters. The SSG group demonstrated significant enhancements in body composition and physical health after the 16-week intervention, including significant improvements in body mass, VO2max, and vertical jump. Conversely, the control group did not experience significant changes in these aspects. These results clearly highlight the potential efficacy of our recreational SSGs program in enhancing body composition and physical health among sedentary young adults.

### 4.1 Effects of recreational SSGs on anthropometric and body composition in sedentary young adults

The recent experimental parallel study revealed that incorporating recreational soccer training in sedentary overweight young adults, both male and female, resulted in a notable enhancement in body composition. These outcomes find congruence with antecedent investigations focusing on sedentary overweight cohorts, such as those conducted by Cvetkovic et al. [14], Soares et al. [39] or Seabra et al. [40]. These studies consistently reported a decrease in body mass index and body fat percentage following participation in recreational soccer, underscoring the effectiveness of recreational soccer in addressing weight-related concerns.

Given that recreational soccer typically involves SSGs, a rationale can be established for the observed enhancements in body composition. These improvements can be ascribed to the high-intensity intermittent nature of the activities, leading to significant caloric expenditure [41]. SSGs often necessitate considerable effort, and while fitted to the participants' abilities, the recurrent high-intensity efforts stemming from individualized participation, heightened demands in acceleration and deceleration, and the dynamic nature of the match in relation to ball possession can account for the discernible impact on participants.

Moreover, SSGs induce a post-exercise reduction in blood glucose concentration [42], and postprandial lipemia [43], facilitating a more favorable adaptation in health parameters and, consequently, body composition. Noteworthy improvements in insulin sensitivity, a pivotal factor in weight management, have been documented in the context of SSGs [44]. Furthermore, the social and team-oriented dimensions inherent in SSGs contribute significantly to enhanced adherence, fostering a positive behavioral environment conducive to long-term engagement.

While additional research is required to comprehensively grasp the underlying physiological and hormonal mechanisms that might elucidate the positive adaptations of anthropometrics and body composition variables to recreational soccer, the efficacy observed in the current study is notably intriguing. This affirmation aligns with previous research [22], creating a compelling narrative that, in conjunction with other non-pharmacological approaches like dietary measures, establishes a robust behavioral foundation for consolidating a healthier lifestyle.

### 4.2 Effects of recreational SSGs on physical fitness in sedentary young adults

Following a 16-week intervention utilizing SSGs, sedentary overweight males and females experienced notable improvements. These positive outcomes were absent in the control groups, thereby underscoring the effectiveness of the current training protocol in augmenting physical fitness parameters within the targeted population.

Enhancements in aerobic performance were evident in both the increased distance covered during the shuttle run test and the estimated maximal oxygen uptake. This observation is consistent with findings from prior studies examining the effects of recreational soccer training programs on overweight sedentary populations, as demonstrated in the works of Hornstrup et al. [45], Seabra et al. [40], Soares et al. [46], and Vasconcellos et al. [47].

Research indicates that SSGs elicit substantial cardiovascular responses, including increased heart rate and oxygen consumption [48], promoting improvements in aerobic fitness over time. The dynamic nature of SSGs necessitates rapid shifts between aerobic and anaerobic energy systems, engaging both oxidative and glycolytic pathways [42]. This metabolic versatility places a demand on the cardiovascular system to deliver oxygen to working muscles efficiently, thereby enhancing the cardiovascular response. Moreover, the variability in movement patterns and intensities during SSGs stimulates the recruitment of diverse muscle groups, fostering a comprehensive physiological adaptation [49]. This multifaceted approach, targeting both cardiovascular and muscular systems, contributes synergistically to the improvements in aerobic performance observed with regular participation in small-sided games.

Engaging in SSGs, particularly those with fewer players and confined spaces, triggers specific neuromuscular adaptations that are associated with heightened lower-limb power as demonstrated previously in recreational soccer [20]. The dynamic nature of SSGs involves swift and repetitive accelerations and decelerations, presenting challenges to the neuromuscular system [50]. These challenges prompt adjustments in muscle recruitment patterns, potentially leading to enhanced efficiency in fiber recruitment.

The specific movement patterns intrinsic to SSGs, characterized by rapid changes in direction and velocity, closely replicate the mechanics of jumping. This replication involves capitalizing on overcoming inertia through force application and, in the case of vertical jumps, exploiting the stretching-shortening cycle [50]. The overload imposed by the acceleration and deceleration demands in SSGs may stimulate physiological responses crucial for enhancing lower-limb power. This includes heightened motor unit recruitment and improved capabilities in force production.

### 4.3 Study limitations, future research and practical applications

The present study has certain limitations. One factor is the absence of alternative experimental training programs (e.g., involving other recreational sports or utilizing analytical approaches) to assess the effectiveness of the current program compared to a similar physical exercise regimen. Another limitation stems from the initially low baseline levels; hence, the incorporation of a high-volume program, as implemented, may have contributed to the observed improvements. Future research should explore comparisons among multiple intervention programs and test various training volumes with the goal of determining the optimal dosage relative to baseline conditions. Additionally, monitoring training load and examining associated changes post-intervention can contribute to a more comprehensive understanding of the mechanisms underlying these improvements.

In terms of practical applications, the current study demonstrates that community-based training programs centered around small-sided games can enhance the physical fitness and body composition of sedentary young adults. Governments and universities may consider establishing facilities and community programs to engage young adults within their own contexts, fostering a community that remains motivated to enhance their health and improve overall well-being and lifestyle. These programs should emphasize offering 2 to 3 training sessions per week, each lasting at least 45 min. During these sessions, participants should be involved in small-sided games that elicit, on average, efforts exceeding 80 % of their maximal heart rate or a perceived exertion rating of 7.5–8 on a 10-point scale. The games should involve a smaller number of players (3v3 to 5v5), utilize small goals (to increase the importance of being close to the finalization zone), and adopt intermittent formats to allow participants to recover actively during periods of exertion.

## 5 Conclusions

This study unveiled that participants engaging in SSGs experienced significant reductions in body mass, BMI, waist circumference, hip circumference, waist-to-hip ratio, and skinfold thickness after the intervention. In contrast, the control group exhibited no significant changes during the observation period. Furthermore, the intervention led to improvements in VO2max as well as running and jumping ability.

These findings suggest that a recreational SSGs intervention has a more pronounced impact on physical fitness parameters among sedentary overweight young adults. Additionally, the intervention holds promise for promoting exercise programs in populations with limited access to sports facilities. Importantly, the study underscores the notion that SSGs can serve as an enjoyable and effective exercise strategy for enhancing physical fitness and improving body composition. Ultimately, this intervention could play a crucial role in safeguarding sedentary overweight populations from the heightened risk of non-communicable diseases associated with obesity and inactivity.

## Data availability

The data is accessible upon request from the corresponding author. Additionally, to ensure replicability, details of the specific exercise dynamics are also obtainable upon request from the corresponding author.

## CRediT authorship contribution statement

**Qi Xu:** Writing – review & editing, Writing – original draft, Resources, Methodology, Investigation, Formal analysis, Data curation, Conceptualization. **Kai Qi:** Writing – review & editing, Writing – original draft, Investigation. **Guiyang Liu:** Writing – review & editing, Writing – original draft. **TingYu Li:** Writing – review & editing, Writing – original draft. **Filipe Manuel Clemente:** Writing – review & editing, Writing – original draft, Visualization, Validation, Supervision, Methodology, Formal analysis, Conceptualization.

## Declaration of competing interest

The authors declare that they have no known competing financial interests or personal relationships that could have appeared to influence the work reported in this paper.

## References

[1] Franco D.C., Ferraz N.L., Sousa T.F.D. (2019). Sedentary behavior among university students: a systematic review. Revista Brasileira de Cineantropometria & Desempenho Humano.

[2] Wang J., Wang Y., Korivi M., Chen X., Zhu R. (2022). Status of sedentary time and physical activity of rural residents: a cross-sectional population-based study in eastern China. Front. Public Health.

[3] Bueno-Antequera J., Munguía-Izquierdo D. (2023).

[4] León-Latre M., Moreno-Franco B., Andrés-Esteban E.M., Ledesma M., Laclaustra M., Alcalde V., Peñalvo J.L., Ordovás J.M., Casasnovas J.A. (2014). Sedentarismo y su relación con el perfil de riesgo cardiovascular, la resistencia a la insulina y la inflamación. Rev. Esp. Cardiol..

[5] Medina C., Tolentino-Mayo L., López-Ridaura R., Barquera S. (2017). Evidence of increasing sedentarism in Mexico City during the last decade: sitting time prevalence, trends, and associations with obesity and diabetes. PLoS One.

[6] Huang Z., Liu Y., Zhou Y. (2022). Sedentary behaviors and health outcomes among young adults: a systematic review of longitudinal studies. Healthcare.

[7] Heine M., Lupton-Smith A., Pakosh M., Grace S.L., Derman W., Hanekom S.D. (2019). Exercise-based rehabilitation for major non-communicable diseases in low-resource settings: a scoping review. BMJ Glob. Health.

[8] Costigan S.A., Eather N., Plotnikoff R.C., Taaffe D.R., Lubans D.R. (2015). High-intensity interval training for improving health-related fitness in adolescents: a systematic review and meta-analysis. Br. J. Sports Med..

[9] Ramos J.S., Dalleck L.C., Tjonna A.E., Beetham K.S., Coombes J.S. (2015). The impact of high-intensity interval training versus moderate-intensity continuous training on vascular function: a systematic review and meta-analysis. Sports Med..

[10] Alzar-Teruel M., Aibar-Almazán A., Hita-Contreras F., Carcelén-Fraile M. del C., Martínez-Amat A., Jiménez-García J.D., Fábrega-Cuadros R., Castellote-Caballero Y. (2022). High-intensity interval training among middle-aged and older adults for body composition and muscle strength: a systematic review. Front. Public Health.

[11] Wewege M., van den Berg R., Ward R.E., Keech A. (2017). The effects of high‐intensity interval training vs. moderate‐intensity continuous training on body composition in overweight and obese adults: a systematic review and meta‐analysis. Obes. Rev..

[12] Milanović Z., Pantelić S., Čović N., Sporiš G., Krustrup P., Milanovic Z., Pantelic S., Covic N., Sporis G., Krustrup P. (2015). Is recreational soccer effective for improving VO2max ? A systematic review and meta-analysis. Sports Med..

[13] Castagna C., de Sousa M., Krustrup P., Kirkendall D.T. (2018). Recreational team sports: the motivational medicine. J. Sport Health Sci..

[14] Cvetković N., Stojanović E., Stojiljković N., Nikolić D., Scanlan A.T., Milanović Z. (2018). Exercise training in overweight and obese children: recreational football and high‐intensity interval training provide similar benefits to physical fitness. Scand. J. Med. Sci. Sports.

[15] Hadjicharalambous M., Zaras N., Apostolidis A., Tsofliou F. (2022). Recreational soccer, body composition and cardiometabolic health: a training-intervention study in healthy adolescents. Int. J. Human Movement Sports Sci..

[16] Clemente F., González-Fernández F., Ceylan H., Silva R., Ramirez-Campillo R. (2022). Effects of recreational soccer on fat mass in untrained sedentary adults: a systematic review with meta-analysis. Hum. Mov..

[17] Bruun D.M., Krustrup P., Hornstrup T., Uth J., Brasso K., Rørth M., Christensen J.F., Midtgaard J. (2014). “All boys and men can play football”: a qualitative investigation of recreational football in prostate cancer patients. Scand. J. Med. Sci. Sports.

[18] Hulton A.T., Flower D., Murphy R., Richardson D., Drust B., Curran K. (2016). Effectiveness of a community football programme on improving physiological markers of health in a hard-to-reach male population: the role of exercise intensity. Soccer Soc..

[19] Clemente F.M., Moran J., Ramirez-Campillo R., Oliveira R., Brito J., Silva A.F., Badicu G., Praça G., Sarmento H. (2022). Recreational soccer training effects on pediatric populations physical fitness and health: a systematic review. Children.

[20] Luo H., Newton R.U., Ma’ayah F., Galvão D.A., Taaffe D.R. (2018). Recreational soccer as sport medicine for middle-aged and older adults: a systematic review. BMJ Open Sport Exerc. Med..

[21] Bray S.R., Born H.A. (2004). Transition to university and vigorous physical activity: implications for health and psychological well-being. J. Am. Coll. Health.

[22] Sarmento H., Manuel Clemente F., Marques A., Milanovic Z., David Harper L., Figueiredo A. (2020). Recreational football is medicine against non‐communicable diseases: a systematic review. Scand. J. Med. Sci. Sports.

[23] Krustrup P., Aagaard P., Nybo L., Petersen J., Mohr M., Bangsbo J. (2010). Recreational football as a health promoting activity: a topical review. Scand. J. Med. Sci. Sports.

[24] Craig C.L., Marshall A.L., Sjostrom M., Bauman A.E., Booth M.L., Ainsworth B.E., Pratt M., Ekelund U., Yngve A., Sallis J.F., Oja P. (2003). International physical activity Questionnaire: 12-country reliability and validity. Med. Sci. Sports Exerc..

[25] Hume P.A., Kerr D.A., Ackland T.R. (2018). Best Practice Protocols for Physique Assessment in Sport.

[26] Cedillo Y.E., Knight R.O., Darnell B., Fernandez J.R., Moellering D.R. (2022). Body fat percentage assessment using skinfold thickness agrees with measures obtained by DXA scan in African American and Caucasian American women. Nutr. Res..

[27] de Menezes Júnior F.J., de Jesus Í.C., Leite N. (2019). Predictive equations of maximum oxygen consumption by shuttle run test in children and adolescents: a systematic review. Revista Paulista de Pediatria.

[28] Léger L., Gadoury C. (1989). Validity of the 20 m shuttle run test with 1 min stages to predict VO2max in adults. J. Canad. Sci. Sport.

[29] España-Romero V., Ortega F.B., Vicente-Rodríguez G., Artero E.G., Rey J.P., Ruiz J.R. (2010). Elbow position affects handgrip strength in adolescents: validity and reliability of jamar, DynEx, and TKK dynamometers. J. Strength Condit Res..

[30] Bosco C., Luhtanen P., Komi P.V. (1983). A simple method for measurement of mechanical power in jumping. Eur. J. Appl. Physiol. Occup. Physiol..

[31] Bogataj Š., Pajek M., Hadžić V., Andrašić S., Padulo J., Trajković N. (2020). Validity, reliability, and usefulness of my jump 2 app for measuring vertical jump in primary school children. Int. J. Environ. Res. Publ. Health.

[32] Samozino P., Morin J.-B., Hintzy F., Belli A. (2008). A simple method for measuring force, velocity and power output during squat jump. J. Biomech..

[33] Gheller R.G., Dal Pupo J., Ache-Dias J., Detanico D., Padulo J., dos Santos S.G. (2015). Effect of different knee starting angles on intersegmental coordination and performance in vertical jumps. Hum. Mov. Sci..

[34] Padulo J., Tiloca A., Powell D., Granatelli G., Bianco A., Paoli A. (2013). EMG amplitude of the biceps femoris during jumping compared to landing movements. SpringerPlus.

[35] Wilson M.T., Macgregor L.J., Fyfe J., Hunter A.M., Hamilton D.L., Gallagher I.J. (2022). Bayesian analysis of changes in standing horizontal and vertical jump after different modes of resistance training. J. Sports Sci..

[36] Guo Y., Bian J., Li Q., Leavitt T., Rosenberg E.I., Buford T.W., Smith M.D., Vincent H.K., Modave F. (2018). A 3-minute test of cardiorespiratory fitness for use in primary care clinics. PLoS One.

[37] Williams N. (2017). The Borg rating of perceived exertion (RPE) scale. Occup Med (Chic Ill).

[38] Vasconcellos F., Seabra A., Farinatti P. (2014). Recreational soccer to prevent cardiovascular risk factors in obese adolescents: effect of weekly frequency. Med. Sci. Sports Exerc..

[39] Soares I.F., Cunha F.A., Vasconcellos F. (2023). Effects of a 12-week recreational soccer program on resting metabolic rate among adolescents with obesity. J. Sci. Sport Exercise.

[40] Seabra A., Katzmarzyk P., Carvalho M.J., Seabra A., Coelho E.S.M., Abreu S., Vale S., Póvoas S., Nascimento H., Belo L., Torres S., Oliveira J., Mota J., Santos-Silva A., Rêgo C., Malina R.M. (2016). Effects of 6-month soccer and traditional physical activity programmes on body composition, cardiometabolic risk factors, inflammatory, oxidative stress markers and cardiorespiratory fitness in obese boys. J. Sports Sci..

[41] Toh S.H., Guelfi K.J., Wong P. (2011). P. a Fournier, Energy expenditure and enjoyment of small-sided soccer games in overweight boys. Hum. Mov. Sci..

[42] Mendham A.E., Duffield R., Marino F., Coutts A.J. (2015). Differences in the acute inflammatory and glucose regulatory responses between small-sided games and cycling in sedentary, middle-aged men. J. Sci. Med. Sport.

[43] Smallcombe J.W., Barrett L.A., Morris J.G., Sherar L.B., Tolfrey K. (2018). Small-sided soccer in school reduces postprandial lipemia in adolescent boys. Med. Sci. Sports Exerc..

[44] Mendham A.E., Duffield R., Coutts A.J., Marino F., Boyko A., Bishop D.J. (2015). Rugby-specific small-sided games training is an effective alternative to stationary cycling at reducing clinical risk factors associated with the development of type 2 diabetes: a randomized, controlled trial. PLoS One.

[45] Hornstrup T., Póvoas S., Helge J.W., Melcher P.S., Fristrup B., Andersen J.L., Møgelvang R., Hansen P.R., Nybo L., Krustrup P. (2020). Cardiovascular and metabolic health effects of team handball training in overweight women: impact of prior experience. Scand. J. Med. Sci. Sports.

[46] da Silva Soares D.B., Shinjo S.K., Santos A.S., de Cassia Rosa de Jesus J., Schenk S., de Castro G.S., Zanoteli E., Krustrup P., da Silva M.E.R., de Sousa M.V. (2022). Skeletal muscle gene expression in older adults with type 2 diabetes mellitus undergoing calorie-restricted diet and recreational sports training - a randomized clinical trial. Exp. Gerontol..

[47] Vasconcellos F., Seabra A., Cunha F., Montenegro R., Penha J., Bouskela E., Nogueira Neto J.F., Collett-Solberg P., Farinatti P. (2016). Health markers in obese adolescents improved by a 12-week recreational soccer program: a randomised controlled trial. J. Sports Sci..

[48] Hammami A., Kasmi S., Farinatti P., Fgiri T., Chamari K., Bouhlel E. (2017). Blood pressure, heart rate and perceived enjoyment after small-sided soccer games and repeated sprint in untrained healthy adolescents. Biol. Sport.

[49] Li T., Xu Q., Wang S., Qi K., Su P., Silva R.M., Sarmento H., Clemente F.M. (2023). Effects of recreational small-sided games from different team sports on the improvement of aerobic fitness in youth sedentary populations: a systematic review. Heliyon.

[50] Madison G., Patterson S.D., Read P., Howe L., Waldron M. (2019). Effects of small-sided game variation on changes in hamstring strength. J. Strength Condit Res..

