# Supplementary material for: Effects of a 16-week recreational small-sided games soccer intervention on body composition and physical fitness in sedentary young adults: A randomized controlled study
Source: Heliyon. 2024 Jan 29;10(3):e25242. doi: 10.1016/j.heliyon.2024.e25242 (PMC10845911; doi:10.1016/j.heliyon.2024.e25242)
Supplement: Multimedia component 1 [file mmc1.docx]

**Supplementary material 1.** Within-participants variation for anthropometric and body composition variables.


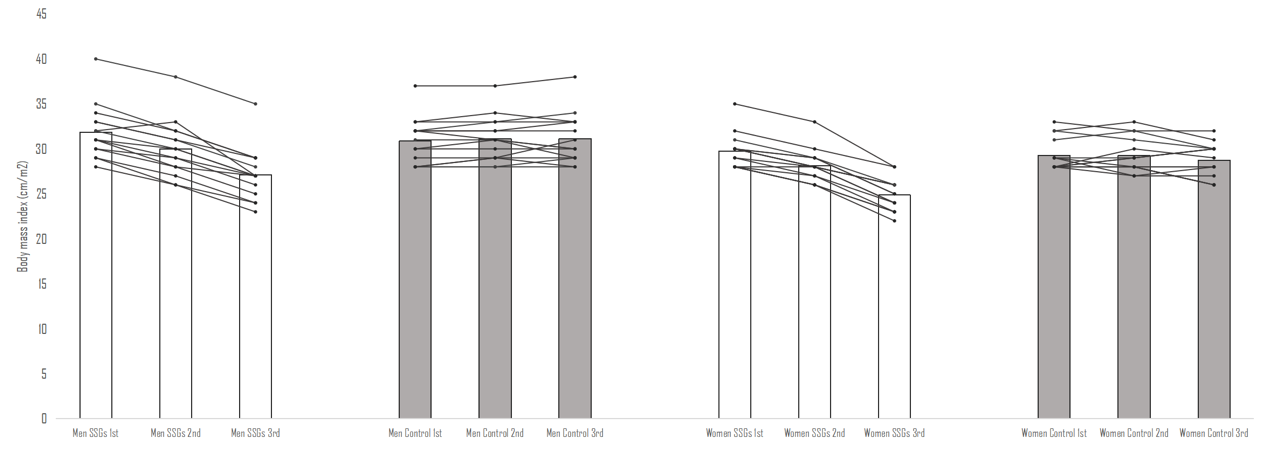


Descriptive statistics (mean and intra-individual variation) of body mass index over the different periods of assessment. White bars: SSGs group; Grey bar: control group)


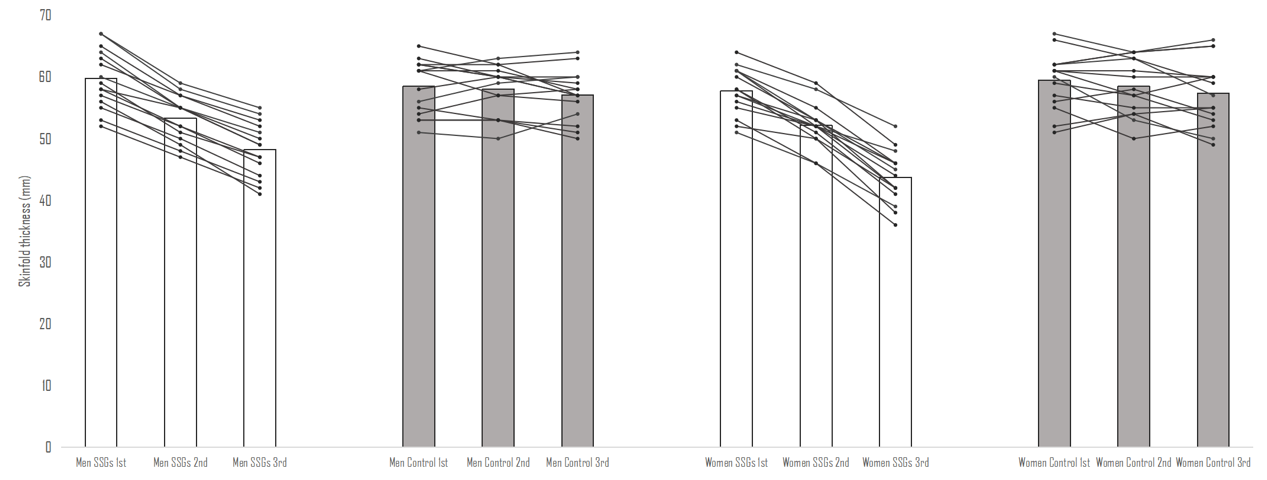


Descriptive statistics (mean and intra-individual variation) of skin fold thickness over the different periods of assessment. White bars: SSGs group; Grey bar: control group)


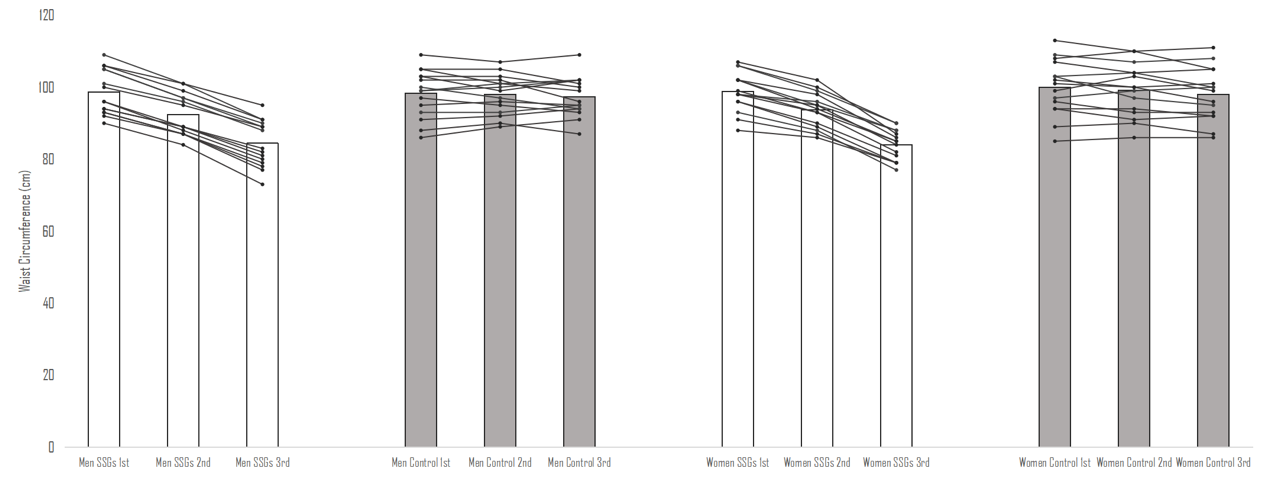


Descriptive statistics (mean and intra-individual variation) of waist circumference over the different periods of assessment. White bars: SSGs group; Grey bar: control group)


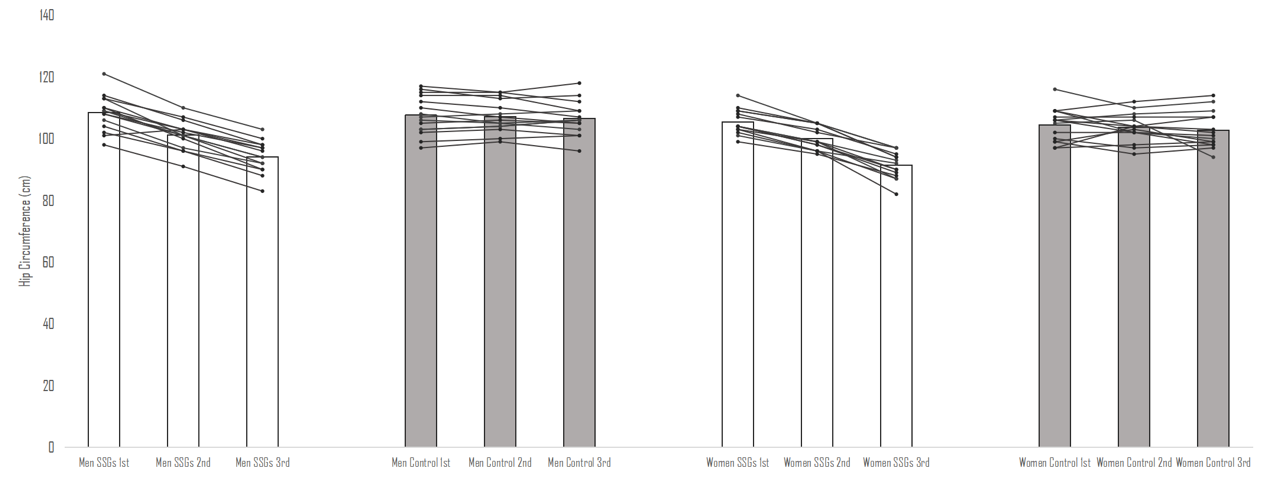


Descriptive statistics (mean and intra-individual variation) of hip circumference over the different periods of assessment. White bars: SSGs group; Grey bar: control group)


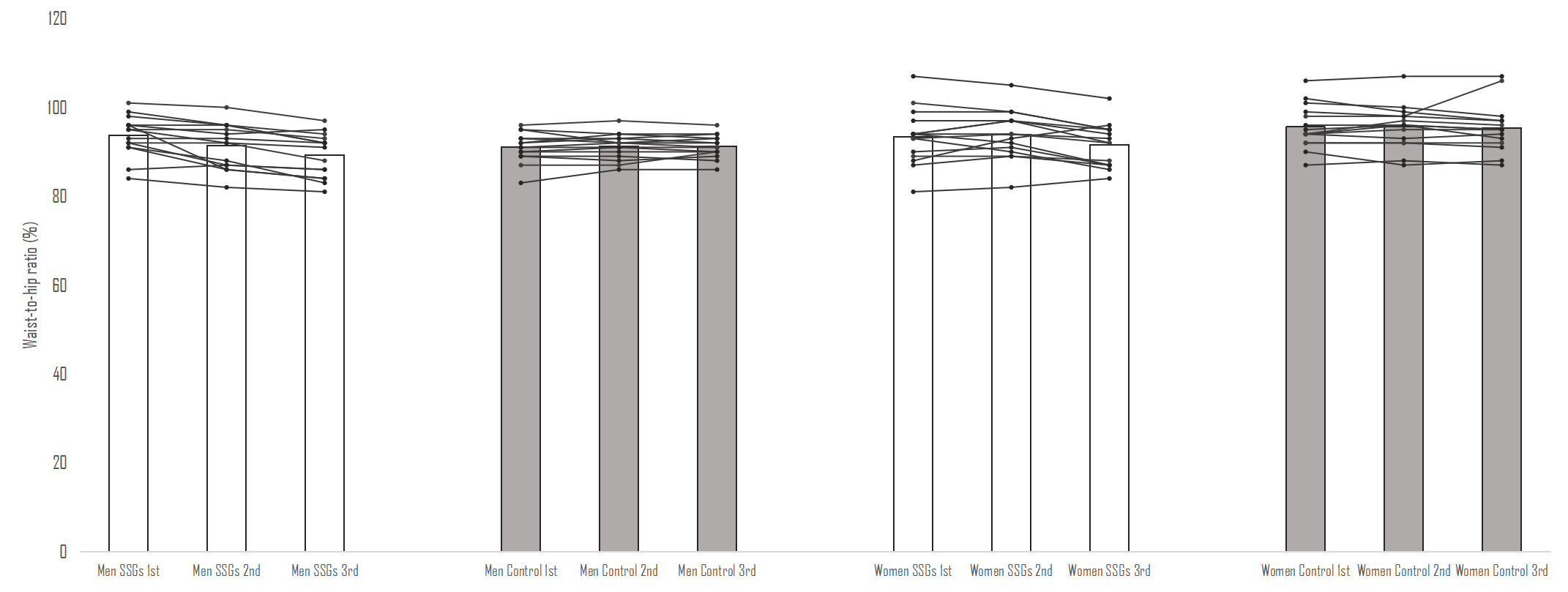


Descriptive statistics (mean and intra-individual variation) of waist-to-hip ratio over the different periods of assessment. White bars: SSGs group; Grey bar: control group)
