# Supplementary material for: Effects of a 16-week recreational small-sided games soccer intervention on body composition and physical fitness in sedentary young adults: A randomized controlled study
Source: Heliyon. 2024 Jan 29;10(3):e25242. doi: 10.1016/j.heliyon.2024.e25242 (PMC10845911; doi:10.1016/j.heliyon.2024.e25242)
Supplement: Multimedia component 2 [file mmc2.docx]

**Supplementary material 2.** Within-participants variation for physical fitness variables.


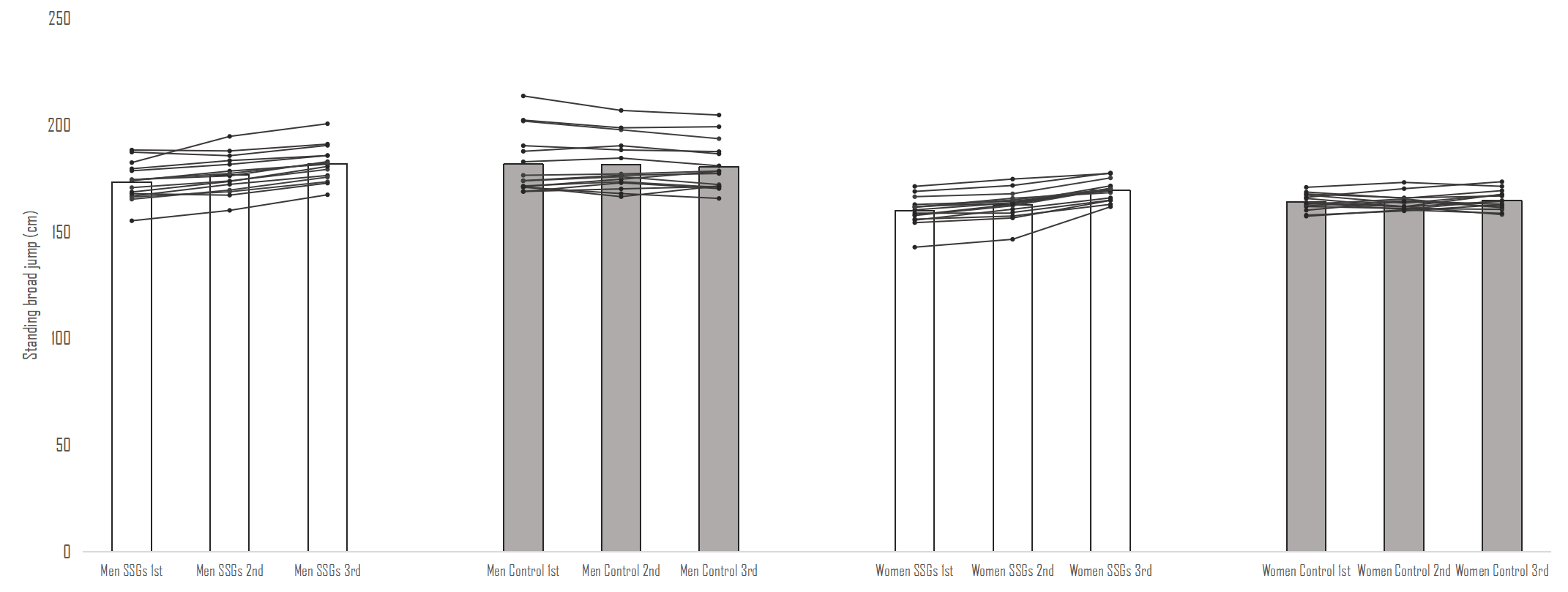


Descriptive statistics (mean and intra-individual variation) of standing broad jump ratio over the different periods of assessment. White bars: SSGs group; Grey bar: control group)


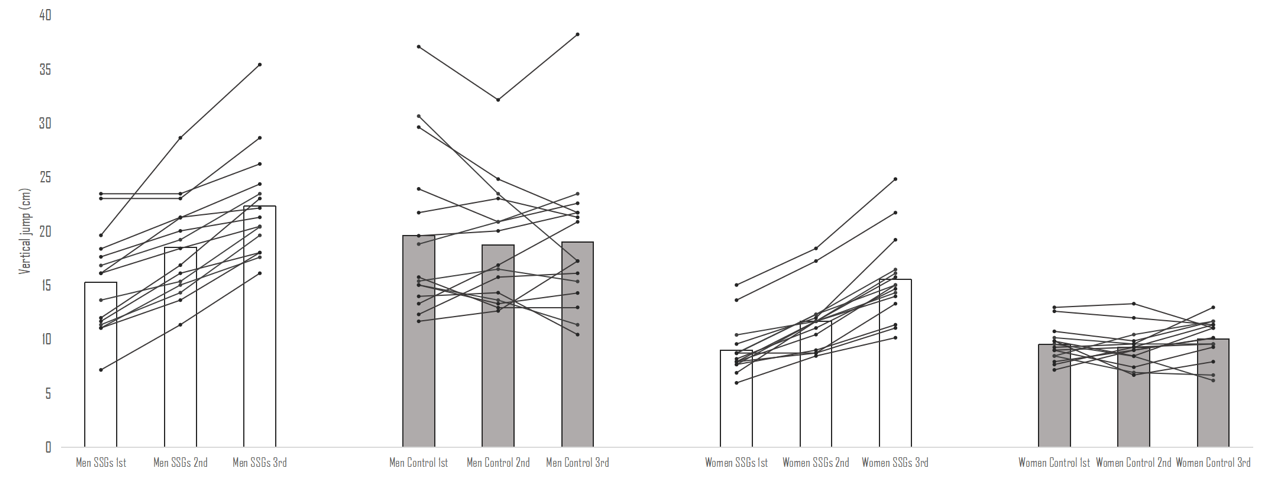


Descriptive statistics (mean and intra-individual variation) of vertical jump over the different periods of assessment. White bars: SSGs group; Grey bar: control group)


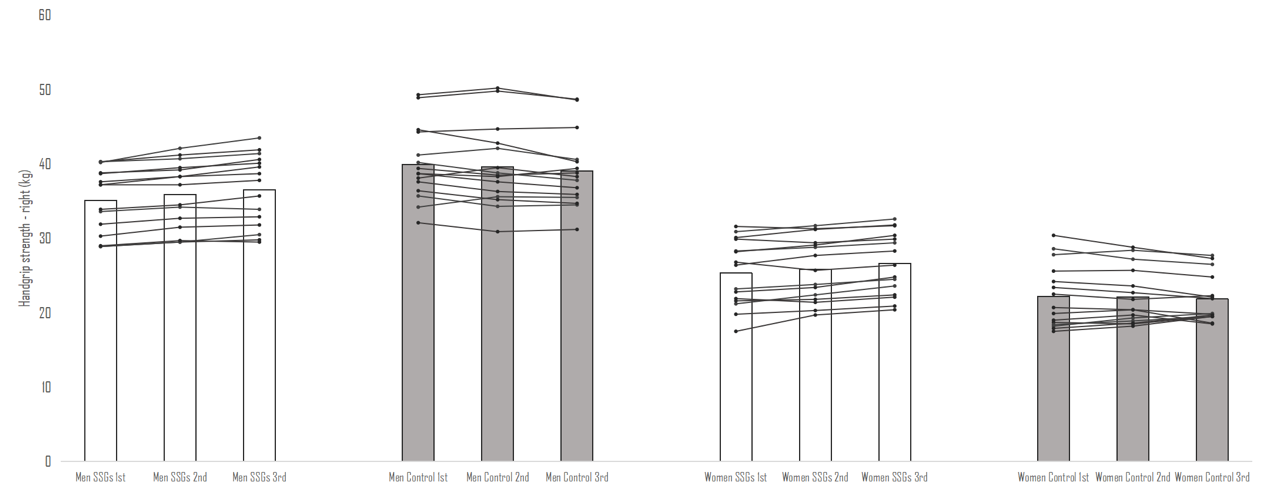


Descriptive statistics (mean and intra-individual variation) of handgrip right over the different periods of assessment. White bars: SSGs group; Grey bar: control group)


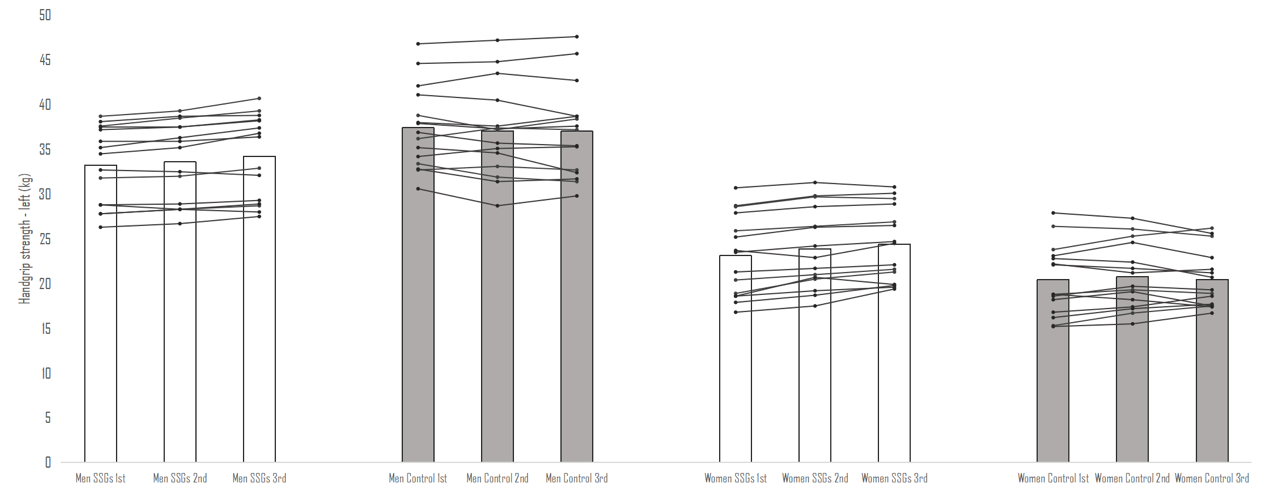


Descriptive statistics (mean and intra-individual variation) of handgrip left over the different periods of assessment. White bars: SSGs group; Grey bar: control group)


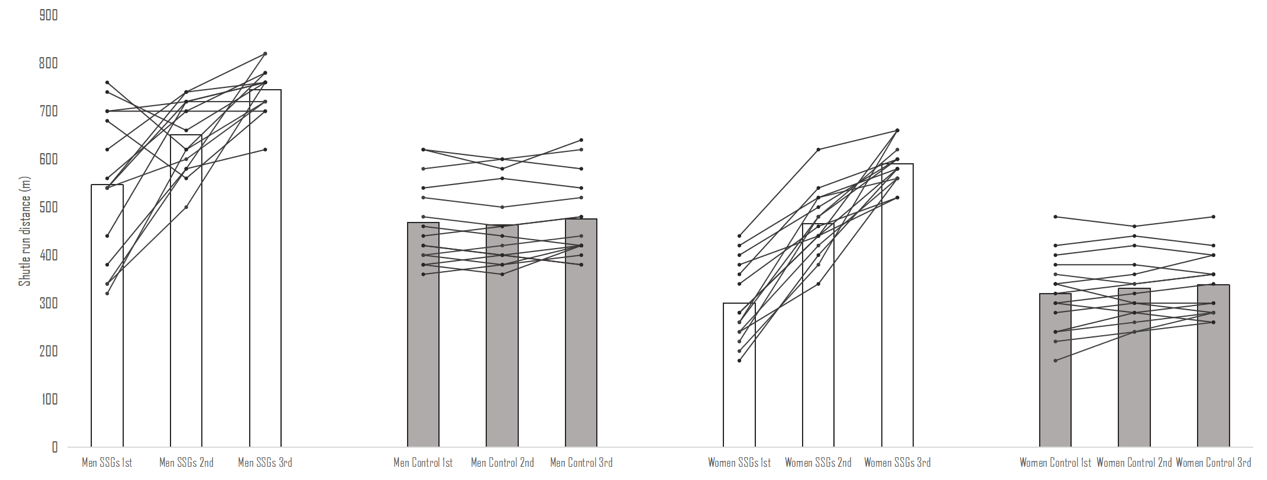


Descriptive statistics (mean and intra-individual variation) of shuttle run distance over the different periods of assessment. White bars: SSGs group; Grey bar: control group)


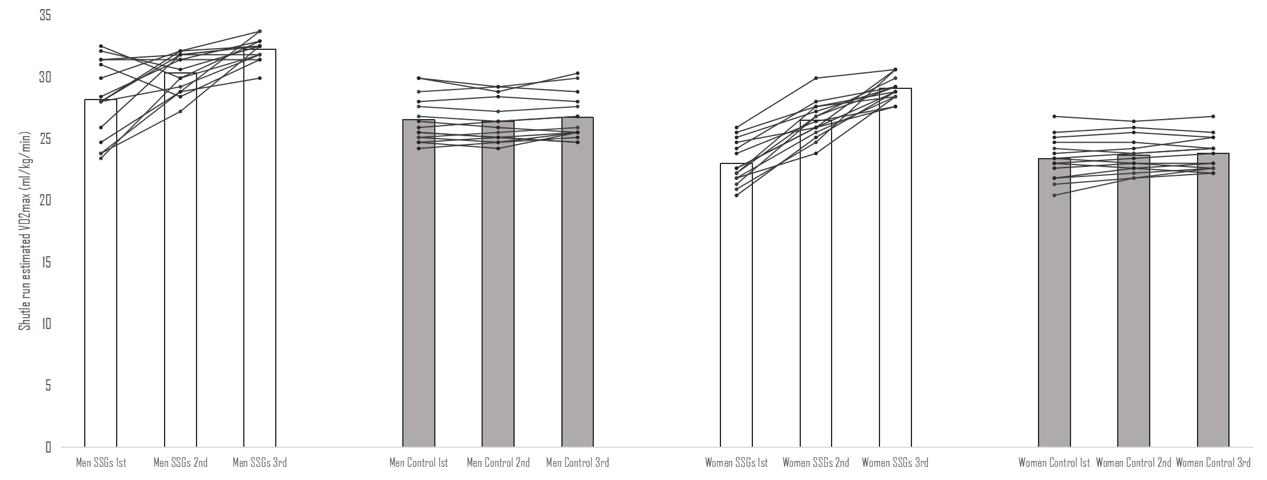


Descriptive statistics (mean and intra-individual variation) of shuttle run estimated VO2max over the different periods of assessment. White bars: SSGs group; Grey bar: control group)
